# Suture tape augmentation in arthroscopic lateral ligament repair for chronic ankle instability yields similar clinical outcomes but faster return to sport compared to isolated repair

**DOI:** 10.1002/ksa.70308

**Published:** 2026-02-06

**Authors:** Pierre‐Henri Vermorel, Jordi Vega, Matteo Guelfi

**Affiliations:** ^1^ Department of Orthopaedic Surgery University Hospital Centre of Saint‐Étienne Saint‐Étienne France; ^2^ Université Jean Monnet Saint‐Etienne, CHU Saint‐Etienne, Lyon 1, Université Savoie Mont‐Blanc, Laboratoire Interuniversitaire de Biologie de la Motricité Saint‐Etienne France; ^3^ Clinica Salus Policlinico di Monza Alessandria Italy; ^4^ Foot and Ankle Unit iMove Traumatology Barcelona Spain; ^5^ MIFAS by Grecmip Merignac France; ^6^ Casa di Cura Villa Montallegro Genoa Italy; ^7^ J Medical Turin Italy

**Keywords:** arthroscopy, chronic ankle instability, internal brace, ligament repair, return to sport

## Abstract

**Purpose:**

Arthroscopic ligament repair is a standard treatment for chronic ankle instability (CAI). In patients with risk factors for failure, isolated repair (ILR) may be insufficient and augmentation techniques have been proposed. This study compared ILR with suture tape–augmented repair (LR + STA), hypothesizing comparable outcomes in higher‐risk patients.

**Methods:**

In this retrospective, indication‐based cohort study, all patients who underwent arthroscopic treatment for CAI between 2019 and 2022 were included. Patients were allocated to two groups: Group A underwent ILR, and Group B underwent LR + STA due to pivoting‐sport participation, generalized ligamentous laxity or poor‐quality remnant ligament. Clinical evaluation was performed preoperatively and at 3, 6, 12 and 24 months using the Foot Function Index (FFI), visual analogue scale (VAS) and Foot and Ankle Ability Measure–Sports Subscale (FAAM‐SS). Return to daily activities (RTD), work (RTW), sport (RTS) and complications were recorded at final follow‐up.

**Results:**

A total of 105 patients (mean age 30.8 ± 14.5 years) were included: 44 in Group A and 61 in Group B. Both groups demonstrated significant improvements in all clinical outcomes from baseline to final follow‐up (*p* < 0.001). At 2 years, no significant between‐group differences were observed in VAS (1.2 ± 1.7 vs. 0.7 ± 1.0; 95% confidence interval [CI]: –1.6; 0.6), FFI (9.7 ± 13.5 vs. 5.7 ± 7.6; 95% CI –12.7; 4.7) or FAAM‐SS (86.6 ± 20.9 vs. 90.5 ± 12.9; 95% CI: –9.9; 17.5). No major complications or recurrences of instability occurred. RTS was significantly earlier in Group B (12 vs. 20 weeks, *p* < 0.001), while RTD and RTW showed no significant differences.

**Conclusion:**

Both isolated repair and suture tape‐augmented repair achieved excellent outcomes with no significant differences at 2 years. Despite being performed in patients with higher functional demands or greater failure risk, suture tape‐augmented repair demonstrated similar complication rates, no recurrence of instability and an earlier RTS.

**Level of Evidence:**

Level III, retrospective cohort study.

**Abbreviations**
ADT: anterior drawer test
ATFL: anterior talo‐fibular ligament
CAI: chronic ankle instability
CFL: calcaneo‐fibular ligament
CRPS: complex regional pain syndrome
FAAM‐SS: Foot and Ankle Ability Measure—Sport Scale
FFI: Foot Function Index
ILR: isolated ligament repair
LFTCL: lateral fibulo‐talo‐calcaneal ligament
LR + STA: ligament repair + suture tape augmentation
MCID: minimal clinically important differences
ROM: range of motion
RTD: return to daily activities
RTS: return to sport
RTW: return to work
VAS: visual analogue scale
VTT: varus talar tilt test

## INTRODUCTION

Chronic ankle instability (CAI) is among the most common traumatic conditions and can significantly affect daily function and quality of life [14, 15, 46]. Although conservative management, including bracing and physiotherapy, is often effective, a considerable number of patients ultimately require surgical intervention [9]. The open Broström procedure has long been considered the gold standard; however, with advances in ankle arthroscopy, arthroscopic lateral ligament repair has gained widespread adoption over the past decade [12, 27, 33]. All‐inside arthroscopic techniques have demonstrated a favourable safety profile and clinical outcomes equivalent to those of open procedures [12, 20, 31, 47], while offering the additional advantage of facilitating a faster return to sport (RTS) [16]. Moreover, arthroscopy provides direct intra‐articular visualization, enabling more accurate diagnosis and allowing concomitant management of associated intra‐articular injuries [8].

In most cases, isolated ligament repair (ILR) is sufficient to restore stability, with a low risk of recurrence [12]. However, evidence suggests that ILR may be insufficient in certain subgroups, including patients with generalized ligamentous laxity, obesity, high functional demands, poor quality of the remnant ligament or significant preoperative laxity, given their higher risk of failure [1, 4, 23, 24, 26, 36, 40, 42].

In such cases, ligament reconstruction (ligamentoplasty) or augmentation of the ligament repair may be recommended, depending on the severity of ankle instability as determined by the ankle instability severity scoring system [2, 9, 13, 23]. In comparison with ligamentoplasty, ligament repair offers several advantages, including accelerated recovery, better outcomes, and the avoidance of graft harvesting, which may be associated with donor site morbidity [9, 32, 41]. When adequate remnant tissue is present, repair, with augmentation when indicated, is generally preferred; reconstruction is reserved for cases with absent or nonviable remnants.

Several techniques have been proposed to augment the ligament repair [4, 38, 39, 40]. Among these options, suture tape‐augmented repair has emerged as a promising alternative [39]. While studies in open surgery have demonstrated that augmentation enhances repair strength, improves joint stability and facilitates faster recovery compared to isolated repair [11, 19, 28], data on arthroscopic techniques incorporating non‐biologic augmentation remain limited [7, 39].

The aim of this study was to compare the clinical outcomes of isolated arthroscopic ligament repair versus suture tape‐augmented repair in a large cohort of patients with CAI.

It was hypothesized that, despite being applied in patients with higher functional demands or risk of failure due to poor ligament remnant quality, suture tape‐augmented repair would achieve clinical outcomes and RTS times comparable to isolated repair.

## METHODS

This retrospective, continuous, monocentric study was conducted in accordance with the principles of the Declaration of Helsinki and received ethical approval from our institutional review board.

All patients who underwent arthroscopic treatment for CAI between 2019 and 2022 were considered for inclusion. Inclusion criteria were skeletally mature patients presenting with CAI for at least 6 months, who had failed a 3‐month course of conservative treatment, including standardized physiotherapy, anti‐inflammatory medication and rest. Exclusion criteria included prior foot or ankle surgery, hindfoot malalignment, ankle osteoarthritis and concomitant tendinous procedure or posterior endoscopy. To ensure a more homogeneous study population, elite athletes training more than three times per week and patients with sedentary office jobs were excluded, as they represent distinct subgroups with either extremely high or extremely low functional demands. Patients were also excluded if a deltoid injury, syndesmosis injury or an osteochondral lesion was identified. Additional exclusion criteria included obesity (body mass index [BMI] ≥ 30), cases of constitutional ligamentous laxity and neuromuscular diseases.

The diagnosis of CAI was established based on patient history, standardized clinical examination and 1.5T magnetic resonance imaging (MRI) findings, all assessed by a single orthopaedic surgeon with expertise in foot and ankle surgery. The clinical history included recurrent ankle sprains or a sensation of giving way for a minimum of 6 months. Clinical examination focused on identifying anterolateral pain, a positive anterior drawer test (ADT) and a varus talar tilt test compared with the contralateral side. MRI evaluation assessed the integrity of the anterior talo‐fibular ligament superior fascicle (ATFL), with or without involvement of the lateral fibulo‐talo‐calcaneal ligament (LFTCL). Finally, arthroscopic evaluation provided definitive characterization of the ligament injury. ATFL superior fascicle injuries were defined as a detachment of the fibular insertion from its footprint. LFTCL injury was diagnosed when the tip of the malleolus was exposed (corresponding to ATFL's inferior fascicle) or when the fibular tendons were visible, indicating involvement of the calcaneo‐fibular ligament (CFL). Baseline data, including sex, BMI and presence of constitutional ligamentous laxity, were also recorded.

### Group assignment

Patients were assigned to two groups based on the procedure performed without randomization: Group A received ILR, whereas Group B received suture tape‐augmented repair (LR + STA). Augmentation was indicated in patients practicing pivoting sports, those with generalized ligamentous laxity (defined by a Beighton score of 5 or higher), combined LFTCL involvement or when poor remnant ATFL's superior fascicle quality was observed during arthroscopy (ATFL's superior fascicle injury Type III–IV) [23, 37]. In all other cases, ILR was performed. The following variables were predefined as potential confounders for adjusted comparisons: age, sex, BMI, operated side, baseline PROs, generalized laxity, remnant ligament quality grade, sport/occupation level and follow‐up duration.

### Clinical and functional evaluation

Clinical evaluations, including the ADT, varus talar tilt test (VTTT), pain on palpation and ankle range of motion (ROM), were performed preoperatively and at the final follow‐up visit. Patient‐reported outcomes, including the visual analogue scale (VAS) for pain, the Foot and Ankle Function Index (FFI) and the Foot and Ankle Ability Measure—Sport Subscale (FAAM‐SS), were prospectively collected preoperatively and at 3, 6, 12 and 24 months using an online database (Surgical Outcome System™; Arthrex). The primary endpoint was prespecified as the FAAM‐SS score at 24 months. Secondary endpoints included FFI, VAS pain, clinical stability tests (ADT and VTTT) and ROM. To contextualize the magnitude of change and between‐group differences, minimal clinically important differences (MCIDs) were defined a priori based on published thresholds: 9 points for FAAM‐SS and 1.5 points for VAS pain [3, 17]. At the latest follow‐up, satisfaction and complications were recorded. The following complications were monitored throughout the entire follow‐up period: recurrent instability, superficial (wound) or deep (joint) infection, persistent pain, ROM limitation and nerve irritation. Recurrent instability was defined as either a subjective complaint of instability or objective instability evidenced by a new episode of ankle sprain. Complications requiring surgical revision (recurrent instability, deep infection, pain persisting >6 months or ROM limitation >10°) were classified as major; all others (superficial infection, nerve irritation, transient pain <6 months or ROM limitation <10°) were considered minor. Each complication was further categorized as transient if it resolved spontaneously before final follow‐up or prior to revision surgery, and as definitive if it persisted.

Return to daily activities (RTD), RTS and return to work (RTW) were also evaluated at each follow‐up using patient self‐reported outcomes. RTS was defined as resumption of the patient's pre‐injury sporting activity, RTW as full resumption of professional duties, and RTD as the ability to perform all pre‐injury activities without limitations.

### Surgical technique

All procedures were performed by a single surgeon specialized in foot and ankle arthroscopy. Arthroscopies were carried out systematically with a dorsiflexion, no‐distraction technique and fully video recorded as part of the patient's permanent medical record.

**Figure 1:**
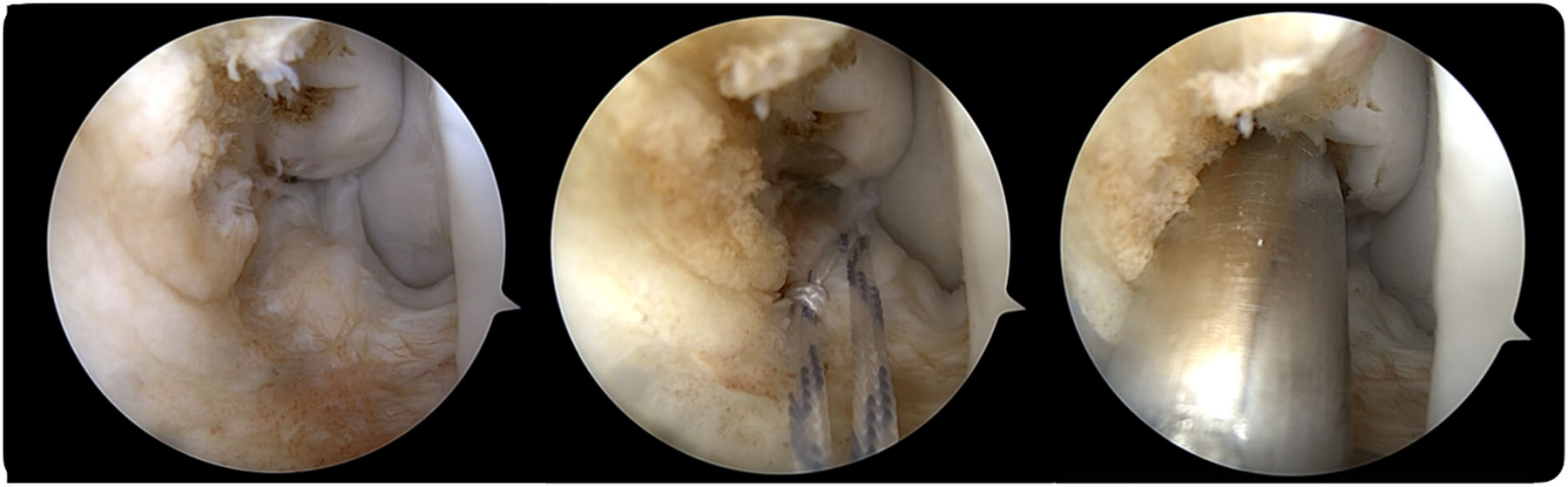
All‐inside isolated repair of ATFL's superior fascicle. The ATFL injury is first identified. Then, the remnant is grasped with a high‐resistance suture, and the tunnel for the anchor is drilled. ATFL, anterior talo‐fibular ligament.

**Figure 2:**
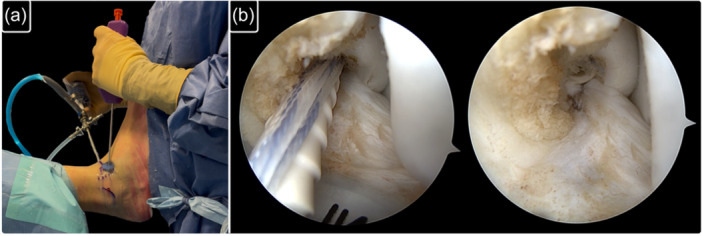
The ATFL is finally reinserted at its footprint using a knotless anchor. (a) External view showing the direction of the anchor. (b) Arthroscopic view of the introduction of the anchor and of the final repair. ATFL, anterior talo‐fibular ligament.

A standardized arthroscopic evaluation of the anterior ankle compartment was conducted following the seven‐point diagnostic protocol previously described in the literature [5, 37]. Concomitant intra‐articular pathologies (soft‐tissue impingement or bony impingement) were addressed first through appropriate debridement. Then, as the last step of the surgery, the lateral collateral ligament repair was performed (Figures 1 and 2). The ATFL's superior fascicle was repaired using a knotless anchor (Pushlock 2.9 × 15 mm, Arthrex) loaded with a high‐resistance suture (Suture Tape, 0.5 mm thick, 1.3 mm wide, Arthrex). In cases with associated LFTCL injury, a small capsular window was created to access the ATFL inferior fascicle and the CFL using a mini‐suture passer. Another high‐resistance suture (#0 FiberWire, Arthrex) was then passed through the LFTCL and loaded in the same anchor than the ATFL's superior fascicle following the original technique [34, 35].

**Figure 3:**
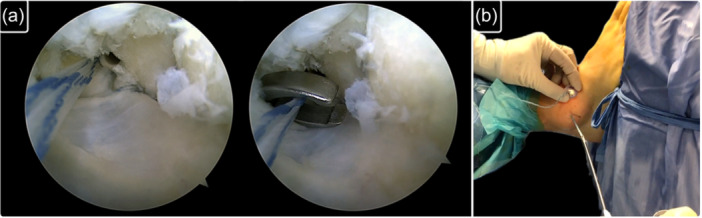
Suture tape augmentation following arthroscopic all‐inside ATFL repair. The suture tape limbs were left attached to the anchor used for the ATFL repair (a). Then, suture remnants are retrieved through the accessory portal (b). ATFL, anterior talo‐fibular ligament.

**Figure 4:**
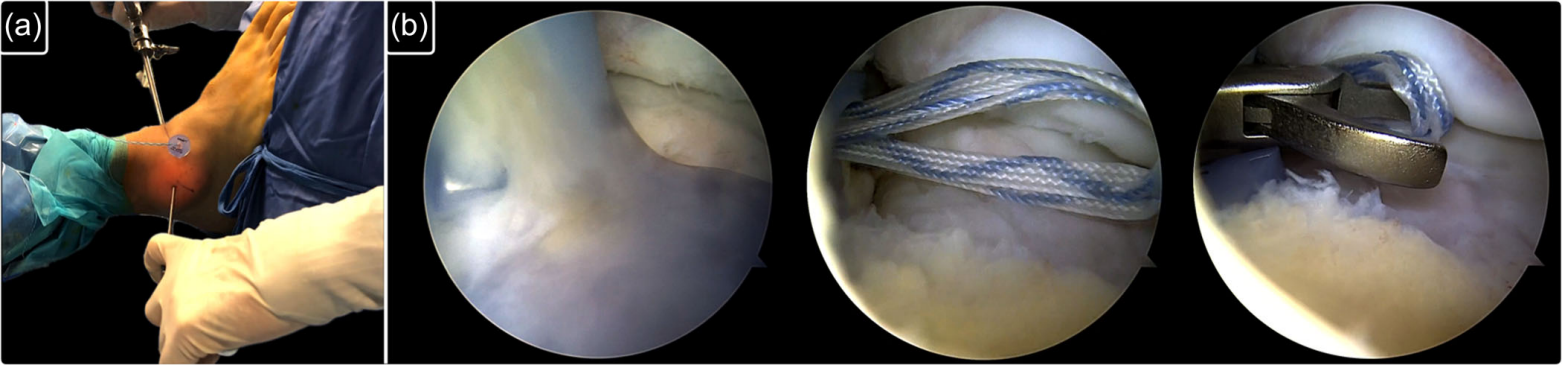
Sutures are subcutaneously passed with a grasper from the accessory portal to the anterolateral portal (a), sliding over the anterolateral ankle capsule using an outside‐to‐inside technique (b). The suture limbs are then pulled out through the anterolateral portal (b).

**Figure 5:**
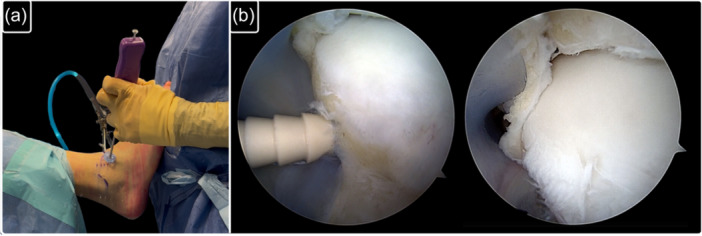
Finally, the suture tape is secured with a knotless anchor placed just anterior to the talar attachment of the ATFL. (a) External view showing the direction of the talar anchor used for the suture tape augmentation. The anchor is directed to the medial malleolus to avoid violation of the subtalar joint. (b) Arthroscopic view of the introduction of the talar anchor used for the augmentation. ATFL, anterior talo‐fibular ligament.

When augmentation was indicated, after ligament repair, the limbs of the suture tape used for ATFL's superior fascicle reinsertion were not cut and were utilized to perform the augmentation, according to the original technique described by Vega et al. [39]. Suture limbs were retrieved from the accessory anterolateral portal (Figure 3). Then, both suture limbs were subcutaneously passed with a grasper from the accessory portal to the anterolateral portal, sliding over the anterolateral ankle capsule in an outside‐to‐inside technique (Figure 4). Finally, the sutures were fixed with a second knotless anchor (Pushlock 2.9 × 15 mm, Arthrex) at the talar ATFL footprint. The tape was tensioned with the ankle in maximal plantarflexion and neutral inversion‐eversion to avoid ROM limitation (Figure 5).

### Postoperative protocol

A standardized postoperative protocol was applied to both groups. Patients wore a removable walking boot for the first 3 postoperative weeks, and full weight‐bearing as tolerated with crutches was permitted beginning on postoperative Day 1. Antithrombotic prophylaxis was administered for 15 days. Physiotherapy also began on the first postoperative day and included active and passive ROM exercises, gait training and muscle strengthening. For patients who underwent ILR, plantarflexion was restricted to 20°, and inversion movements were prohibited during the initial 3‐week period. Proprioceptive and weight‐bearing balance training began at 3 weeks. RTS was authorized once patients had regained full ROM, strength and neuromuscular control.

### Statistical analysis

Return‐to‐activity outcomes (RTD, RTS and RTW) were compared between Groups A and B by analysing the time to return. Other continuous clinical outcomes (VAS, FFI and FAAM‐SS) were compared at each follow‐up interval. For each variable, the distribution within each group was assessed using the Shapiro–Wilk test. When both groups satisfied normality assumptions (*p* > 0.05), data were summarized as mean ± standard deviation, and between‐group differences were analysed using an independent *t* test. When normality was not met, data were reported as median [interquartile range], and comparisons were performed using the Mann–Whitney *U* test. Statistical significance was defined as *p* < 0.05. All analyses were conducted using Python (version 3.x) with the SciPy and pandas libraries.

## RESULTS

**Figure 6:**
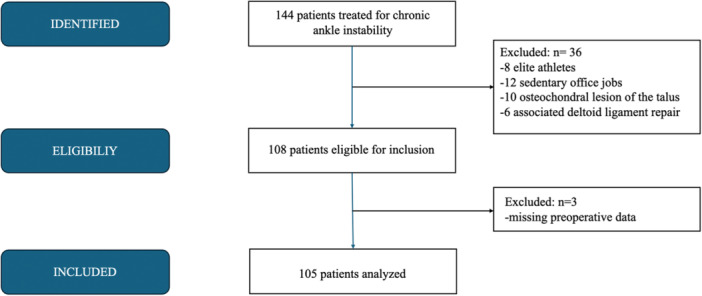
Flowchart.

During the study period, 144 patients underwent arthroscopic treatment for CAI. Of these, 105 patients met the inclusion criteria and were included in the final analysis (Figure 6). The mean age was 30.8 ± 14.5 years (range: 16–75). The cohort included 57 males (54%) and 50 females (46%). The right ankle was involved in 40 cases (38%) and the left in 65 cases (62%). Forty‐four patients were allocated to Group A (ILR) and 61 to Group B (LR + STA). In Group A, all patients presented with an isolated injury of the ATFL superior fascicle. In Group B, 49 patients (80.3%) also had isolated involvement of the ATFL superior fascicle, while 8 patients (13.1%) demonstrated injury to the ATFL inferior fascicle and 4 patients (6.6%) showed additional involvement of the CFL. The two groups were comparable in baseline characteristics, except for gender distribution. Further demographic and clinical details are provided in Table 1.

**Table 1:** Demographic and clinical characteristics of both groups.

	Group A (ILR)	Group B (LR + STA)	*p* value
Population (*n*)	44	61	
Age (years)	33.4 ± 14.2	29.6 ± 14.6	n.s.
Gender (male/female)	20/24	37/24	<0.05
Side (right/left)	16/28	24/37	n.s.
Mean BMI (kg·m^−^^2^)	23.7 ± 5.6	24.2 ± 4.9	n.s.
Mean follow‐up (days)	516 ± 115	485 ± 123	n.s.

*Note*: *p* > 0.05.

Abbreviations: BMI, body mass index; ILR, isolated repair; LR, ligament repair; n.s., nonsignificant; STA, suture tape augmentation.

### Clinical and functional outcomes

Both groups showed significant improvements in FFI, VAS and FAAM‐SS compared with preoperative values (*p* < 0.001). In Group A, mean VAS decreased from 4.8 ± 2.7 (range: 0–8.2) to 1.2 ± 1.7 (range: 0–5.3), a difference of –3.9 (95% confidence interval [CI]: –4.9 to –3.0). Mean FFI improved from 39.9 ± 16.4 (range: 15.2–83.9) to 9.7 ± 13.5 (range: 0–49.1), a difference of –32.1 (95% CI: –37.5 to –26.8). Mean FAAM‐SS increased from 35.9 ± 20.7 (range: 0–90.6) to 86.6 ± 20.9 (range: 31.3–100), a gain of 52.7 (95% CI: 46.9 to 58.4). In Group B, mean VAS decreased from 4.0 ± 2.7 (range: 0–9.0) to 0.7 ± 1.0 (range: 0–3.1), a reduction of –3.8 (95% CI: –4.7 to –2.9). Mean FFI improved from 36.4 ± 15.6 (range: 5.0–63.2) to 5.7 ± 7.6 (range: 0–23.5), a difference of –29.2 (95% CI: –36.2 to –22.2). Mean FAAM‐SS increased from 36.4 ± 15.6 (range: 5.0–63.2) to 90.5 ± 12.9 (range: 62.5–100), an improvement of 47.2 (95% CI: 35.9 to 58.4). There were no statistically significant differences between groups at any intermediate time point for any of the three scores (VAS, FFI and FAAM‐SS) (Table 2).

**Table 2:** Description of clinical and functional outcomes by group over time.

Time point	Group A (ILR)	Group B (ILR + STA)	95% CI	*p* value
VAS				
Preop	4.8 ± 2.7	4.0 ± 2.7	[−2.2; 0.5]	0.22
3 months	1.3 ± 1.4	1.1 ± 1.1	[−1.0; 0.5]	0.26
6 months	1.2 ± 1.1	1.2 ± 1.5	[−0.8; 0.8]	0.46
1 year	0.9 ± 1.2	0.6 ± 1.5	[−1.2; 0.7]	0.31
2 years	1.2 ± 1.7	0.7 ± 1.0	[−1.6; 0.6]	0.37
FFI				
Preop	39.9 ± 16.4	36.4 ± 15.6	[−11.8; 4.8]	0.18
3 months	14.3 ± 11.5	13 ± 10.7	[−5.5; 8.0]	0.36
6 months	9.9 ± 9.2	8.3 ± 7.2	[−3.6; 6.7]	0.3
1 year	6.1 ± 3.8	5.6 ± 6.8	[−4.1; 3.1]	0.42
2 years	9.7 ± 13.5	5.7 ± 7.6	[−12.7; 4.7]	0.42
FAAM SS				
Preop	35.9 ± 20.7	36.4 ± 15.6	[−9.4; 10.4]	0.47
3 months	68.8 ± 19.6	70.3 ± 22.7	[−15.0; 11.9]	0.4
6 months	82.1 ± 15.4	84.4 ± 13.8	[−11.7; 7.2]	0.33
1 year	89.2 ± 10.9	89.7 ± 11.2	[−7.4; 8.4]	0.16
2 years	86.6 ± 20.9	90.5 ± 12.9	[−9.9; 17.5]	0.17
RTD (weeks)	13 [6–14]	12 [6–18]	NA	0.67
RTS (weeks)	20 [12–24]	12 [6–12]	NA	<0.001
RTW (weeks)	12 [6–12]	12 [6–18]	NA	0.29

*Note*: Normally distributed variables are presented as mean ± standard deviation, whereas non‐normally distributed variables are reported as median [interquartile range].

Abbreviations: BMI, body mass index; CI, confidence interval; FAAM‐SS, Foot and Ankle Ability Measure–Sports subscale; FFI, Foot Function Index; ILR, isolated repair; LR, ligament repair; NA, not available; n.s., nonsignificant; STA, suture tape augmentation; VAS, visual analogue scale.

No cases of recurrent ankle instability or major complications requiring revision surgery were reported in either group. Transitory complications occurred in 2 patients (4.5%) in Group A and 4 patients (6.5%) in Group B, with no significant difference between groups. In Group A, one patient developed a complex regional pain syndrome (CRPS) Type I solved within 6 months, and one experienced a transitory dysesthesia in the territory of the superficial peroneal nerve. In Group B, four patients reported persistent anterolateral pain at 3 months; all were successfully managed with intra‐articular corticosteroid injections. Of the 105 patients, 101 reported being satisfied or very satisfied with their procedure, while four patients (three in Group B and one in Group A) expressed dissatisfaction.

At the final follow‐up, all patients reported subjective improvement in ankle stability. Clinical examination showed negative ADT and VTTT in every case. All patients had resumed daily and recreational activities without limitation, and no significant ankle ROM deficit was observed compared with the contralateral side.

### RTW, RTS and RTD

At the last follow‐up, all patients returned to their daily life activity, sport or work. No significant differences were observed between groups regarding RTD (respectively, 13 [6, 7, 8, 9, 10, 11, 12, 13, 14] vs. 12 [6, 7, 8, 9, 10, 11, 12, 13, 14, 15, 16, 17, 18] weeks, *p* = 0.67) and RTW (respectively, 12 [6, 7, 8, 9, 10, 11, 12] vs. 12 weeks [6, 7, 8, 9, 10, 11, 12, 13, 14, 15, 16, 17, 18], *p* = 0.29). However, patients in Group B returned to sport significantly earlier than those in Group A (20 [12, 13, 14, 15, 16, 17, 18, 19, 20, 21, 22, 23, 24] vs. 12 [6, 7, 8, 9, 10, 11, 12, 13, 14, 15, 16, 17, 18], *p* < 0.001) (Table 2).

## DISCUSSION

The most important finding of this study is that both isolated repair and suture tape‐augmented repair achieved excellent and comparable functional outcomes. However, patients who underwent suture tape augmentation returned to sport significantly earlier than those treated with isolated repair, whereas the time to resume daily activities and RTW did not differ between groups. Notably, the suture tape augmented group included patients with higher functional demands or risk of failure due to poor ligament remnant quality. These findings suggest that suture tape augmentation provides additional protection to the ligament repair and facilitates functional recovery.

Both isolated repair and suture tape‐augmented repair significantly improved all clinical (VAS) and functional outcomes (FFI, FAAM‐SS) at the 2‐year follow‐up compared with preoperative values, achieving excellent mean scores. These results are consistent with previous literature supporting the effectiveness of arthroscopic repairs for CAI [12, 20, 36, 39, 40]. The absence of major complications or revision surgeries in both groups reinforces the safety of these procedures and supports the concept of arthroscopic repair as the current standard of care [33].

Although patients in the LR + STA group represented a higher‐risk cohort, characterized by greater laxity, poor remnant ligament quality or participation in pivoting sports, no significant differences were observed in FFI, FAAM‐SS, VAS or complication rates at any follow‐up. In addition, the LR + STA group demonstrated a significantly shorter RTS interval, suggesting potential advantages in patients with high activity demands.

This advantage may be explained by the internal brace‐like support of the synthetic augmentation, which protects the repair and permits earlier and more intensive rehabilitation with a reduced risk of repair rupture [21, 28, 29, 30, 43]. In contrast, the time to RTW and daily activities did not differ significantly between groups, likely reflecting the lower physical demands of these activities, which may mask differences in functional recovery. These findings are in line with the literature that reports excellent outcomes after both biological and non‐biological augmentation [2, 29].

Some studies suggest that outcomes and recurrence of ankle instability after ILR may be influenced by the quality of the remnant ligaments [18, 45]. A recent study reported inferior clinical outcomes, particularly in sports‐related activities, along with higher recurrence rates in cases with poor‐quality remnants compared with good‐quality tissue [18]. Our results suggest that suture tape augmentation may help address this limitation and improve the outcomes of ligament repair in patients with poor‐quality remnant ligaments.

Lopes et al. recommend ligamentoplasty in such cases [22, 23], though this approach is associated with longer recovery, inferior functional outcomes, higher intraoperative complication rate and increased donor‐site morbidity compared with repair [10, 32, 41]. Furthermore, preoperative prediction of ligament quality is challenging, leaving the definitive choice between repair and ligamentoplasty to intraoperative assessment. Suture‐tape augmentation may therefore represent the recommended treatment for patients with poor‐quality remnants, enhancing repair strength [29].

Previous studies have shown that patients with generalized ligamentous laxity and high functional demands have an increased risk of failure [22, 23, 24, 26]. Park et al. reported a failure rate of up to 45% in patients with generalized ligamentous laxity undergoing ILR [26]. For this reason, Lopes et al. recommended ligament reconstruction in these high‐risk patients [23]. In our study, however, patients with such high‐risk factors achieved excellent outcomes without failures when treated with ligament repair augmented with suture tape. These findings suggest that augmented repair is an effective treatment option for these patients, representing a valuable alternative to ligamentoplasty. Further comparative studies are needed to directly evaluate both techniques in high‐risk patients.

Concerns have been raised that synthetic augmentation may overconstrain the joint and restrict ROM, particularly in plantarflexion [44]. To minimize this risk, suture tape was fixed in plantarflexion in all patients. At final follow‐up, no differences in ROM were observed between the operative and contralateral sides. Additionally, the first generation of non‐biological sutures used in anterior cruciate ligament reconstruction was associated with severe synovial inflammatory reactions in the 1990s [6], leading to skepticism regarding intra‐articular synthetic grafts. Although newer generations of non‐biological sutures appear to have overcome this limitation [25], our technique deliberately positions the augmentation extra‐articularly, over the joint capsule, to further reduce the risk of inflammatory response. Nonetheless, persistent anterolateral pain was reported at 3‐month follow‐up in four patients of the LR + STA group, which may hypothetically reflect a localized foreign‐body reaction. Further studies are required to clarify the potential for inflammatory responses associated with suture augmentation. However, in all cases, the pain was successfully managed conservatively.

The excellent clinical outcomes of LR + STA, with comparable complication rates and faster recovery than isolated repair, might encourage its routine use in all cases of ankle instability. However, every surgical procedure carries a potential risk of complications, and in the authors' opinion, a systematic augmentation may increase the risk of surgical complications, such as anterolateral pain. Given the excellent results of ILR when appropriately indicated, this additional risk appears unnecessary.

This study has several limitations. Its retrospective design introduces potential selection and information biases, although prospective data collection likely mitigated these risks to some extent. The type of sport and occupation was not recorded; these unmeasured confounders may influence recovery and therefore limit the interpretation of return‐to‐activity timelines. To reduce this bias and improve group homogeneity, patients with extremely high functional demands (elite athletes) or very low demands (sedentary workers) were excluded. Group allocation was not randomized, as the indication for internal brace augmentation depended on patient‐specific factors such as activity level, degree of laxity and quality of the remnant ligament. Although clinically relevant, this introduces heterogeneity and limits direct comparability between groups. A randomized controlled trial would more definitively evaluate the effectiveness of suture‐tape augmentation. The rehabilitation protocol was also faster and more intensive in the suture tape‐augmented group, which could be viewed as a potential confounder; however, this accelerated progression was intentionally permitted due to the additional mechanical support provided by the internal brace and should be considered an inherent advantage of the technique rather than a source of bias. Additionally, this is a single‐surgeon series, which may limit external generalizability. Finally, although the cohort size was relatively large (*n* = 105), the study may still have been underpowered to detect rare complications.

The main clinical relevance of this study is that suture tape‐augmented repair achieved excellent outcomes comparable to isolated repair, with similar complication rates, despite being performed in patients with higher functional demands or risk of failure due to poor ligament remnant quality. In addition, suture tape‐augmented repair facilitated an earlier RTS, suggesting that arthroscopic all‐inside repair with augmentation may be particularly valuable in patients at increased risk of failure after isolated repair or in those requiring rapid RTS.

Considering our results, while acknowledging the limitations of this study, suture‐tape augmented repair appears most appropriate in patients with a Beighton score ≥5, those presenting a combined injury of the ATFL superior fascicle and the CFL, or those in whom poor tissue quality of the ATFL superior fascicle is identified arthroscopically (injury Types III–IV). Further randomized controlled trials comparing suture‐tape augmentation with isolated repair or reconstruction in this specific population are needed to validate this proposed treatment algorithm.

## CONCLUSION

Both arthroscopic isolated repair and suture tape‐augmented repair achieved excellent and comparable outcomes in the treatment of ankle instability at 2‐year follow‐up. Despite being performed in patients with higher functional demands or risk of failure due to poor ligament remnant quality, suture tape augmentation demonstrated similar complication rates, no recurrence of instability and an earlier RTS. In view of the nonrandomized allocation, these findings warrant confirmation in randomized studies specifically targeting this high‐risk population.

## AUTHOR CONTRIBUTIONS

**Pierre‐Henri Vermorel:** statistical analysis, manuscript drafting. **Jordi Vega:** study conception and design, critical manuscript revision. **Matteo Guelfi:** data collection, study conception and design, critical manuscript revision.

## CONFLICT OF INTEREST STATEMENT

The authors declare no conflict of interest.

## ETHICS STATEMENT

This study was conducted in accordance with the principles of the Declaration of Helsinki and received approval from our Institutional Review Board (IRB number 2021‐09‐020).

## Data Availability

The research data supporting this study are not publicly shared.
